# The diversity and commonalities of the radiation-resistance mechanisms of *Deinococcus* and its up-to-date applications

**DOI:** 10.1186/s13568-019-0862-x

**Published:** 2019-09-03

**Authors:** Mengmeng Jin, Anqi Xiao, Liying Zhu, Zhidong Zhang, He Huang, Ling Jiang

**Affiliations:** 1 College of Biotechnology and Pharmaceutical Engineering, Nanjing Tech University, Nanjing, 210019 People’s Republic of China; 2 College of Food Science and Light Industry, Nanjing Tech University, Nanjing, 210009 People’s Republic of China; 3 College of Chemical and Molecular Engineering, Nanjing Tech University, Nanjing, 210009 People’s Republic of China; 4 Institute of Microbiology, Xinjiang Academy of Agricultural Sciences, Urumqi, Xinjiang Uigur Autonomous Region People’s Republic of China; 5 College of Pharmaceutical Science, Nanjing Tech University, Nanjing, 210009 People’s Republic of China

**Keywords:** *Deinococcus*, Ionizing radiation, DNA repair, Anti-oxidation

## Abstract

*Deinococcus* is an extremophilic microorganism found in a wide range of habitats, including hot springs, radiation-contaminated areas, Antarctic soils, deserts, etc., and shows some of the highest levels of resistance to ionizing radiation known in nature. The highly efficient radiation-protection mechanisms of *Deinococcus* depend on a combination of passive and active defense mechanisms, including self-repair of DNA damage (homologous recombination, MMR, ER and ESDSA), efficient cellular damage clearance mechanisms (hydrolysis of damaged proteins, overexpression of repair proteins, etc.), and effective clearance of reactive oxygen species (ROS). Due to these mechanisms, *Deinococcus* cells are highly resistant to oxidation, radiation and desiccation, which makes them potential chassis cells for wide applications in many fields. This article summarizes the latest research on the radiation-resistance mechanisms of *Deinococcus* and prospects its biotechnological application potentials.

## Introduction

Extremophilic microorganisms have a wide range of potential applications due to their high resistance to extreme environments. *Deinococcus* is one of the most radiation-resistant extremophiles in the world, tolerating up to 15,000 Gy of acute ionizing radiation and 60 Gy/h of chronic radiation (Daly 2006). What’s more, its capacity to withstand ionizing radiation is 1000 times that of typical eukaryotes, more than 250 times that of *Escherichia. coli*, and 3000 times that of humans (Cox and Battista 2005; Makarova et al. 2001). In addition, the resistance of *Deinococcus* to drought and hypertonic stress is also relatively high. Therefore, *Deinococcus radiodurans* has been studied widely since it was discovered, and has even become a research hotspot in recent years, both in China and abroad. Its radiation-resistance mechanism has been described, and some studies identified the genes responsible for its radiation-resistance capacity and introduced them into other microorganisms through genetic engineering, so as to increase their application range. In recent years, *D. radiodurans* has been investigated as a platform for the bioremediation of contamination with radiation or heavy metals, and the treatment effect was found to be better than using less tolerant microorganisms.

## Basic properties of *Deinococcus*

When *D. radiodurans* was first isolated from radiation-sterilized corned beef cans by Anderson et al. (Duggan et al. 1963) in1956, it was thought to be affiliated with *Micrococcus* due to morphological similarities. After in-depth research, researchers later classified it into its own family and genus, *Deinococcaceae* and *Deinococcus*. Generally, as shown in Additional file 1: Table S1, *Deinococcus* is a heterotrophic, non-pathogenic, non-motile, non-spore-forming, aerobic tetracoccus (Maisch et al. 2012). It develops red or pink, smooth colonies on TGY medium (0.5% tryptone, 0.3% yeast extract, 0.1% glucose, 1.5–2% agar) after 2–3 days of culture at 30 °C.

The cell envelope of *D. radiodurans* is thick, which is why most cells stain Gram-positive, but it contains two membranes separated by a peptidoglycan layer, which makes it more similar to typical Gram-negative bacteria. Some strains of *Deinococcus* have a cell envelope composed of six layers. The innermost layer is the cell’s inner membrane, which is composed of unusual lipids, including alkylamine chains, followed by a perforated peptidoglycan cell wall, after which there are unique small compartments. The fourth layer is the outer plasmalemma, the fifth layer is composed of different electroluminescent regions, and the sixth layer is composed of hexagonal protein subunits (Gerber et al. 2015). The whole tetrad is surrounded by a dense carbohydrate shell, which contributes to the biological robustness of *Deinococcus*. *Deinococcus* has a robust and unique structure, with cells often forming tetrads (Cox and Battista 2005, Gerber et al. 2015; Ghosal et al. 2005). It also has a unique genomic structure, and the condensed genome can reduce nucleic acid damage when subjected to external stress (ionizing radiation, UV radiation, oxidation, drying, mitomycin C, etc.).

The analysis and annotation of related gene sequences showed an abundant genetic and adaptive diversity of radiation-resistant microorganisms in radiation-contaminated areas of China, and also showed that there are a large number of unknown functional gene resources awaiting discovery in these radiation-contaminated areas, which provides scientific materials and a theoretical basis for further utilization of these genetic resources. The currently known 69 strains of *Deinococcus* as well as their characteristics are summarized in Additional file 1: Table S1. In general, *Deinococcus* are aerobic, non-motile, non-spore forming and non-pathogenic bacteria that grow as red or pink colonies on plates, and mostly stain as Gram-positive in spite of a double membrane. The genus *Deinococcus* has high resistance to γ-radiation, UV radiation, desiccation and mitomycin C, and colonizes a wide range of habitats, including animals and plants, sandy beaches, oceans, the air, deserts, hot springs, high-radiation areas, cold polar regions, etc. (Additional file 1: Table S1). *D. Radiodurans* R1 was the first strain to be discovered with a resistance to γ-radiation and UV radiation, and is a model strain for use in biological research. The complete genome sequence of R1 consists of two chromosomes (2648,638 and 412,348 bp), a megaplasmid (177,466 bp), and a small plasmid (45,704 bp), and its G +C content is 66.6% (White 1999).

## Resistance mechanisms of *Deinococcus*

**Fig. 1:**
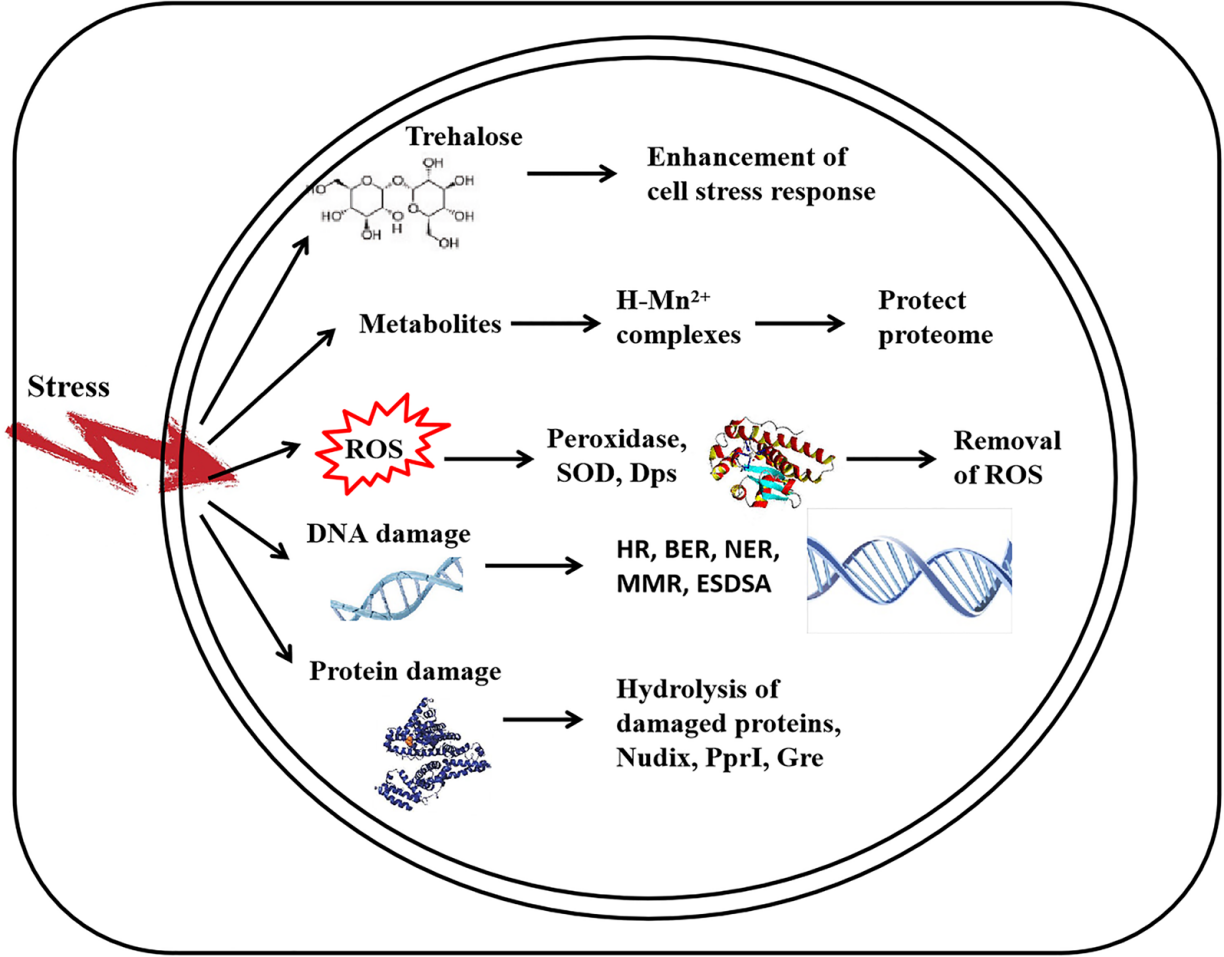
Radiation-resistance mechanisms of *Deinococcus*

As shown in Fig. 1, *Deinococcus* has a systematic radiation-resistance mechanism. Ionizing radiation (IR) can produce reactive oxygen species (ROS) and cause double-stranded DNA breaks (DSBs). Because of the DNA protection mechanism, the genomes of *D. radiodurans* are tightly linked and form ring-like nuclei under IR. The level of DNA damage in *D. radiodurans* and *E. coli* cells induced by IR is similar, but the specific nucleus-like structure in *D. radiodurans* helps to keep the DNA ends formed by the double-strand break together and promote their repair. Compared to *E. coli* cells, which die with 12 double stranded DNA breaks, the DNA repair system of *D. radiodurans* is highly efficient and can successfully repair up to 200 double strand breaks without reducing the cell- viability. Daly (Daly 2009) put forward a view that protein is an important macromolecule substance affected by IR. *D. radiodurans* accumulates manganese complexes (Daly et al. 2004) when exposed to IR, which can prevent the production of iron-dependent reactive oxygen species, thereby protecting the activity of enzymes that repair the DNA. Another theory suggested that IR resistance is predominantly a metabolic phenomenon (Sharma et al. 2017). In this view, IR-resistant cells contain a high cellular content of Mn^2+^ in high-symmetry (H) antioxidant complexes (H-Mn^2+^) with small metabolites, and the complexes (H-Mn^2+^) protect the proteome rather than the genome from IR damage. Additionally, a cross-kingdom analysis of the differences in taxonomic classification, genome size, and radioresistance between cell types, indicated that small, highly symmetric antioxidant complexes of manganese ions and metabolites (H-Mn^2+^) are responsible for cellular IR resistance, not DNA repair systems and antioxidant enzymes (Sharma et al. 2017). The combined action of various mechanisms and evolution of *D. radiodurans* have enabled the bacteria to resist IR.

### Efficient repair of DNA damage

#### Homologous recombination (HR)

The multiple copies of the genome of *Deinococcus* enable efficient repair of double-strand breaks by homologous recombination. Homologous recombination is the main way to repair DNA damage. It uses normal and intact homologous DNA as template to repair damaged DNA, both of which are double-stranded DNA molecules. One of the most important steps in homologous recombination is the interaction between RecA protein and single stranded DNA in areas where double strands were broken to produce free 3′ ends by the RecBCD or RecFOR system in bacteria. Because there are no RecB and RecC proteins in *Deinococcus*, the RecFOR system plays a major role in DNA terminus processing (Agapov and Kulbachinskiy 2015). RecN is an adhesin-like chromosome structure maintenance protein and its ATPase activity stimulates RecA to invade homologous DNA strands to form D-loop structures and repair broken double-stranded DNA. Correspondingly, the binding of RecA with DNA can also promote the ATPase activity of RecN protein (Uranga et al. 2017). Single-stranded DNA binding protein (SSB) protects single-stranded DNA (ssDNA) from degradation and the migration rate of the SSB protein of *D. radiodurans* on single-stranded DNA is one order of magnitude faster than that of the SSB of *E. coli* (Kim et al. 2015). The exonuclease RecJ is essential in *D. radiodurans*, and deletion of the *recJ* gene is lethal. The function of RecQ helicase is replaced by the UvrD helicase, and UvrD helicase has a wide range of functions in *D. radiodurans*, notably in the late stage of nucleotide excision and replication. Recent studies on UvrD have shown that it can unwind DNA in the 3′ → 5′and 5′ → 3′ directions, and the latter activity is influenced by SSB (Agapov and Kulbachinskiy 2015). The newly formed ssDNA eventually interacts with SSB protein and the RecFOR complex, after which RecA binds to the DNA, and a new nucleic acid chain is synthesized by DNA polymerase using intact homologous DNA as template for repair. The highly conserved *recF*-*dr1088* operon was identified in DR1088 by Kaiying Cheng et al. (Cheng et al. 2017), and DR1088 showed single/double stranded DNA binding activity, ssDNA binding protein (SSB) substitution ability and ssDNA annealing activity. Furthermore, *dr1088* is crucial for cell viability, and deleting it directly results in growth defects and increased sensitivity to gamma and UV radiation to different degrees.

#### Extended Synthesis-Dependent Strand Annealing (ESDSA)

Under ionizing radiation, the genome of *D. radiodurans* breaks into a large number of DNA fragments which are partially homologous to each other, and can be used to synthesize new DNA strands, after which long-linear DNA intermediates are transformed into circular genome replication intermediates by RecA to complete the repair (Schmier et al. 2017; Slade and Radman 2011). RecA-mediated homologous recombination involves RecF and RecR proteins, with roles in RecA activation and DNA stabilization, respectively, and these recombination processes initiate the ESDSA pathway in *D radiodurans* (Satoh et al. 2012). DdrB is a radiation-induced specific protein of *Deinococcus* and it has in vitro characteristics similar to SSB protein and can promote the annealing of single-stranded DNA. Previous studies have shown that DdrB can stimulate the annealing of complementary single-stranded DNA in vitro, and its deletion increases the lag period of the annealing process of extended synthesis-dependent strands, and affects the efficiency of DNA synthesis and recombination (Bouthier et al. 2011). In addition, DdrB can help accurately assemble a large number of small fragments generated by extreme radiation through single-stranded annealing (SSA), and generate suitable substrates for the subsequent ESDSA pathway. The mechanism by which the DdrB protein of *D. radiodurans* assists in the precise annealing of single-stranded DNA has been elucidated and described as a “restricted access two-step” process, which has two main phases. In the first phase, DdrB limits the search for complementarity to a subset of bound bases, and in the second stage, the buried bases are checked for additional complementarity in the opposing strand. In addition, DdrB-ssDNA, a single-stranded annealing protein, binds ssDNA in an extended state along a continuous surface of the protein’s oligomeric loop to confirm that ssDNA does not form a secondary structure at high energy, thereby improving annealing accuracy (Sugiman-Marangos et al. 2016).

#### Excision repair

Excision repair includes base excision repair (BER) and nucleotide excision repair (NER). BER corrects small lesions in the DNA double helix structure caused by spontaneous decay, deamination, oxidation or methylation of DNA. BER is initiated by DNA glycosylases, which cleave the bonds between deoxyribose and modified or mismatched DNA bases. Together, these enzymes initiate base excision and repair of a large number of base lesions, each of which is recognized by one or more overlapping specific DNA glycosylases. The DNA glycosylases recognize and remove damaged bases, leaving a base-free site that is further processed by short or long patch repair, which involve different proteins (Krokan 2013). UvrABC and UvsE are the two main NER systems of *Deinococcus*. UvsE protein, induced by ultraviolet radiation, is a Mn^2+^ dependent endonuclease with specificity for pyrimidine dimers, and the UV damage endonuclease (UvsE)-dependent excision repair (UVER) pathway can effectively remove cyclobutane pyrimidine dimers (CPDs) and pyrimidine (6-4) pyrimidine photoproducts (6-4PPs) from genomic DNA (Tanaka et al. 2005).

#### DNA mismatch repair (MMR)

DNA mismatch repair in *D. radiodurans* preferentially repairs deletion mutations rather than insertion mutations for two reasons. One may be related to the recombination bias caused by the polyploid nature of the *D. radiodurans* genome or the result of evolution. The second reason is related to the size of the mutant fragment and the chromosome region in which it is located (Long et al. 2018), which is a unique repair method for this bacterium. Reactive oxygen species (ROS) cause the conversion of guanine to 8-oxo bridge guanine, thereby causing a transversion mutation of GC-AT. The uracil produced during cytosine deamination is efficiently recognized and eliminated by uracil-DNA glycosylases (UDGs). *D. radiodurans* contains four enzymes of the UDGs family, three of which are active, which enhances the clearance of false uracil bases, thereby reducing the probability of C to T mutations (Long et al. 2015).

### Efficient cellular mechanisms

#### Hydrolysis of damaged proteins

Protein degradation includes different cellular responses to environmental stimuli and removal of potentially toxic damaged proteins or protein aggregates. ATP-dependent proteases play a key role in these processes and they are involved in processing of proteins, which includes key regulatory factors. Therefore, they play an important role in various stress reactions, enabling bacteria to survive DNA damage, heat shock or ROS. Under stress conditions, the proteolytic activity of *Deinococcus* cells is greatly enhanced, which is beneficial to the removal of damaged and misfolded proteins. This function is related to the Lon protease system and the ClpXP protease encoded by the *D. radiodurans* genome. The highly conserved Clp protease is a two-component enzyme that contains protein hydrolysis subunits and ATPase subunits with catalytic sites. The ATPase subunits mediate specific hydrolysis reaction by binding substrate proteins and transferring them to the catalytic sites. By contrast, in the Lon protease family, proteolytic enzymes and ATPase activities are present in the same polypeptide (Servant et al. 2007). Servant et al. (2007) found that the inactivation of ClpPX protease significantly reduced the cell survival rate with the increase of gamma-irradiation dose, and the inactivation of Lon1 and Lon2 proteases reduced the resistance to purinomycin, suggesting that they play an important role in eliminating damaged proteins. Some proteins, such as translation factors, serine proteases and β and β’ subunits of RNA polymerase, can avoid degradation (Joshi et al. 2004), which may be essential for rapid recovery of cellular function after radiation stress.

#### Nudix hydrolase

*Deinococcus* (Awile et al. 2010) has a group of proteins containing significant intrinsically disordered regions that are not present in non-extremophile homologues and Nudix hydrolase is one of these proteins with low-complexity N-terminal and C-terminal tails. It removes the diphosphate group from damaged nucleoside triphosphates and prevents their incorporation into DNA. Nudix hydrolases show intrinsic disordered regions with unknown functions and these intrinsic disordered regions increase the surface properties of the folded regions they connect, making them more hydrophilic as a whole, enabling them to interact in this way. By studying the disorder tendency of Nudix hydrolase encoded by the UniProtKB Q9RW5_DEIRA sequence (DRNH), researchers (Awile et al. 2010) found that it has disorder-inducing amino acids (such as glycine and proline) and extremely hydrophilic polar amino acids (such as arginine and lysine), all of which are the decisive factors of internal sequence disorder. The substrates of Nudix hydrolase are cytosine 5′-diphosphate (CDP) and cytosine 5′-triphosphate (CTP), and the reaction requires the participation of bivalent metal cations (Buchko et al. 2008). The genome of *D. radiodurans* encodes 23 Nudix hydrolases, 5 of which are induced by ionizing radiation (Liu et al. 2003). Damaged nucleoside monophosphates can be further dephosphorylated and removed from the cells.

#### PprI

Specific protein PprI (IrrE) is a broad-spectrum transcription factor and a unique regulatory protein, which stimulates *recA* gene transcription after exposure to ionizing radiation (Earl et al. 2002). PprI regulates protein synthesis, including that of stress proteins involved in DNA repair, such as PprA, RecA and SSB. PprI can bind the promoter regions of *recA* and *pprA*, and the protein hydrolysis activity of PprI depends on Mn^2+^ (Wang et al. 2015). However, the mechanism by which PprI regulates gene expression is still unclear, and no protease substrates have been identified. Nevertheless, the lack of this factor significantly reduces the cells’ resistance to ionizing and ultraviolet radiation and to mitomycin C. Wen et al. (2016) constructed the eukaryotic expression vector pEGFP-c1-*pprI* and stably integrated the *pprI* gene into human lung epithelial cell line BEAS-2B, which enhanced their radiation resistance, reduced the rate of gamma-H2AX foci formation and apoptosis in irradiated BEAS-2B cells, and alleviated radiation-induced G2/M blockage. In addition, they transferred the pEGFP-c1-*pprI* vector into the muscles of BALB/c mice by electroporation. It was found that the expression of *pprI* reduced the damage to the hematopoietic system, lung, small intestine and testis induced by acute radiation and increased the survival rate of irradiated mice by regulating the expression of Rad51 in different organs. Chen et al. (2017) injected the plasmid containing the *pprI* gene into the muscles of mice exposed to gamma-ray radiation of 6 Gy and the *pprI* gene was transfected into cells by electroporation in vitro. It was found that the expression of *pprI* gene significantly reduced the mortality rate of mice exposed to lethal doses of irradiation, alleviated the acute phase effect in blood cells, shortened the duration of lymphocyte depletion, and reduced the apoptotic rate of spleen cells, thymocytes and lymphocytes. PprI accelerates the repair of radiation-induced DNA damage by regulating the expression of DNA repair genes and enhances the activity of antioxidant enzymes. Gre is also an important family of transcription factors, represented by GreA and two Gre factor homologues (Gfh1 and Gfh2) in *Deinococcus*. The family-specific Gfh protein binds to the secondary channel of RNA polymerase (RNAP), which enhances the site-specific transcription pause and is closely related to Mn^2+^ and PprI protein (Esyunina et al. 2016).

#### The removal of ROS

Reactive oxygen species (ROS) are by-products of water irradiation and in an extreme atmosphere, more ROS is produced in cells. Free oxygen radicals can destroy DNA, RNA and proteins, thus threatening fundamental cellular processes and survival. The scavenging capacity of ROS reflects the antioxidant capacity of cells. The genome of *Deinococcus* encodes two peroxidases, three catalases (CAT), four superoxide dismutases (SOD) and two Dps proteins (Slade and Radman 2011; Zeng et al. 2017). Taken together their activity against ROS is tens of times higher than that of *E. coli*. Dps is a functional protective protein that binds to Fe^2+^ and oxidizes it to Fe^3+^, avoiding producing excess radicals, and it also has a high binding affinity for DNA, thus preventing hydroxyl radicals from destroying DNA. A high proportion of Mn^2+^/Fe^2+^ inside cells can help them resist oxidative damage (Zeng et al. 2017). A high proportion of Mn^2+^ can help remove ROS, but Fe^2+^ can cause enormous cell damage. The interaction between Mn^2+^ and *D. radiodurans* proteins as well as peptides is responsible for protecting proteins from ROS damage during irradiation. In addition, Peana et al. (2018) predicted the Mn^2+^ binding proteins encoded by the *D. radiodurans* genome, and made similar predictions for other bacteria. The results showed that in most cases, the content of Mn^2+^ binding proteins in radiotolerant bacteria was significantly higher than that of radiation-sensitive bacteria.

The carotenoids found by our team in R12 are also a class of antioxidant metabolites. The whole genome sequencing of a new species of *Deinococcus, Deinococcus wulumuqiensis* R12, was recently completed (Xu et al. 2013; Wang et al. 2009). Three ORFs, orf01490, orf00123 and orf00124, with similarities of 85%, 87% and 91%, were identified by BLAST comparisons between the genomic sequences of *D. wulumuqiensis* R12 and the lycopene synthesis genes of the model strain *D. radiodurans* R1. Based on these sequences, 6 recombinant bacterial strains (pET-EBI, pET-EIB, pET-BEI, pET-BIE, pET-IEB, pET-IBE) were constructed by tandem expression of these three genes (*crtE*, *crtB*, *crtI*), and the lycopene synthase gene cluster of *D. wulumuqiensis* R12 was successfully cloned. Subsequently, through the optimization of gene arrangement and the construction of highly efficient strains through expression engineering, the fermentation of the strains reached maximum yields of 688 ± 10 mg/L (Xu et al. 2018a, b, c). The recombinant bacterium EDW11 was constructed by tandem expression of these three genes and through the optimization of the upstream SD regions and the culture medium, the strain EDW11 produced 88 mg lycopene/g dry cell wt (780 mg lycopene/L) after 40 h fermentation without IPTG induction (Jin et al. 2015). Additionally, the recombinant *E. coli* 99DH with a yield of up to 925 mg/L lycopene was obtained by comparing the yield of lycopene in different media (Xu et al. 2018a, b, c). A large number of stress-resistance genes and genes for the biosynthesis of natural products were found via sequencing, which laid a foundation for further verification of microbial radiation-resistance mechanisms and functional product mining through comparative genomics.

#### Stress metabolite of trehalose

**Fig. 2:**
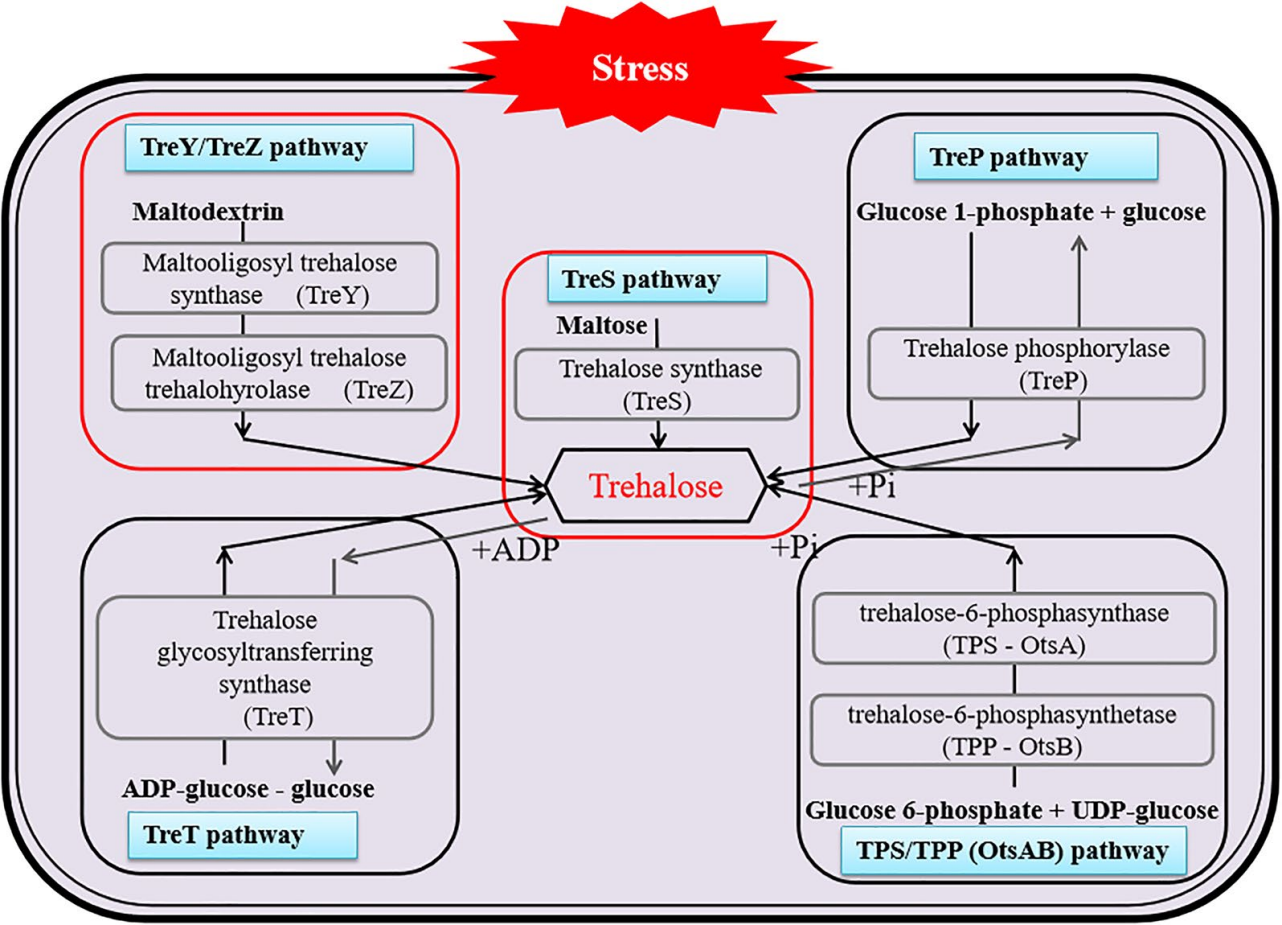
Microbial cells synthesize trehalose under stress. There are five pathways for trehalose synthesis, TPS/TPP, TreY/TreZ, TreS, TreP, and TreT pathway. *Deinococcus* contain the TreY/TreZ and TreS pathways (Jiang et al. 2013; Filipkowski et al. 2012; Panek et al. 2013; Xu et al. 2013; Wang et al. 2009)

Trehalose is a natural stable non-reducing sugar, which is composed of two glucose molecules connected through an α,α-(1,1) glycosidic bond. It is a compatible solute and stress metabolite in cells. Its chemical properties are stable and its content in certain organisms can be as high as 20% of the dry cell weight. Studies have shown that trehalose can be used as a structural component in organisms (e.g. as a basic component of trehalose lipids, participating in the synthesis of cell walls), and directly provide energy, as well as act in transport, signaling or regulation (Elbein et al. 2003). A schematic representation of the synthesis pathway of trehalose is shown in Fig. 2. There are five pathways for trehalose synthesis, TPS/TPP (Elbein 1974; Sugimoto 1995; Ohtake and Wang 2011), TreY/TreZ, TreS, TreP (Han et al. 2003), and TreT (Nobre et al. 2008; Qu et al. 2004; Ryu et al. 2005) pathway. The TreY/TreZ and TreS pathways exist in *Deinococcus* (Jiang et al. 2013; Filipkowski et al. 2012; Panek et al. 2013; Xu et al. 2013; Wang et al. 2009). TreY/TreZ encodes Maltooligosyl trehalose synthase (MTSase) and Maltooligosyl trehalose trehalohyrolase (MTHase), respectively. This pathway is firstly catalyzed by MTSase to produce maltooligosyl-trehalose, which is then hydrolyzed by MTHase to form trehalose (Maruta et al. 1996). Trehalose synthase (TreS) converts maltose directly into trehalose by converting the α,α-1,4 glycosidic bond to the α,α-1,1 glycosidic bond (Jiang et al. 2013; Wang et al. 2007; Wei et al. 2004).

Trehalose synthesis is regulated by the DNA repair switch gene *pprI* (encoding the global transcription regulatory protein IrrE) and universal pressure response factor RpoS (Zhao et al. 2015). At the same time, it can improve the activity of antioxidant enzymes, reduce the accumulation of ROS and inhibit lipid peroxidation (Schluepmann et al. 2003). It is also a regulator of glycometabolism, whereby it can affect the activity of key enzymes such as hexokinase to avoid glycolysis overflow (Gerber et al. 2015). In addition, trehalose affects the mitochondrial oxidative phosphorylation metabolism, and mediates the formation of the cAMP-CRP complex to regulate RpoS and related reactions (Noubhani et al. 2009). As a signal molecule, trehalose regulates interactions between lipids and proteins to stabilize the cell membrane structure, affects transmembrane ATPase activity and modifies the activity of protein kinases (Gläfke et al. 2012). Therefore, as a metabolite produced by cells under stress conditions, trehalose can improve the stress response of cells by regulating a series of metabolic pathways (such as the intake of carbon sources, transcriptional regulation, electron transfer, energy metabolism, protein folding and cell membrane structure). Thus, it is an important stress-resistance factor of great research value in the evolution and adaptation of microbes to environmental stress.

### Applications of *Deinococcus*

#### Removal of heavy metal ions

**Fig. 3:**
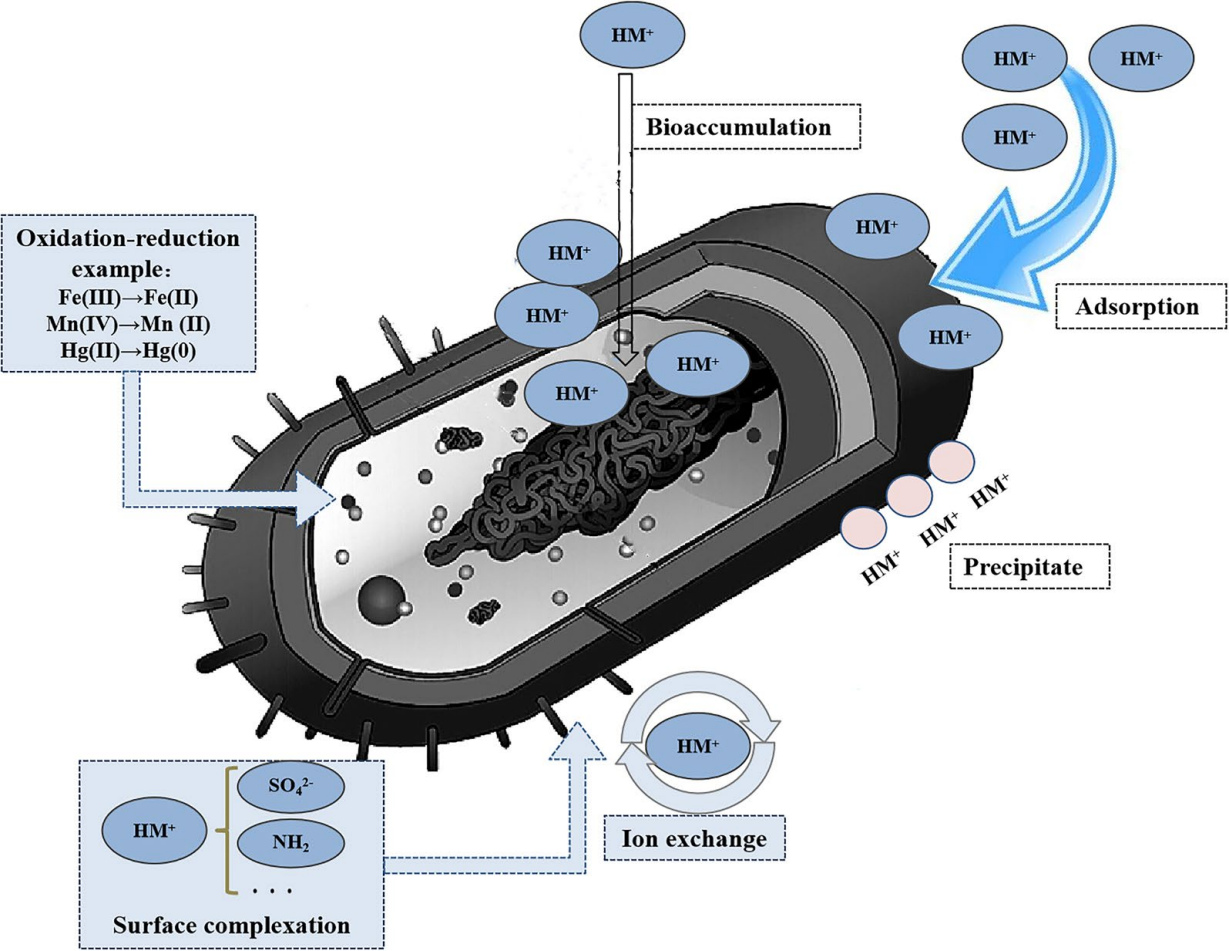
Mechanisms for the microbial treatment of heavy metal ions

Environmental pollution and its effective prevention and control have always been a global topic, and soil pollution has become increasingly serious. Heavy metals mainly refer to cadmium, chromium, mercury, lead, arsenic and other highly toxic metals. In addition, they also encompass heavy metal ions with certain toxicity such as copper, cobalt, zinc, nickel, tin, vanadium and so on. Soil heavy metals have poor mobility, long residence times and are difficult to remove from the soil, which makes their remediation very challenging. In the production processes of machinery manufacturing, smelting industry, chemical industry, electronics and other industries, wastewater rich in heavy metal ions is often produced, and these heavy metals can be enriched in the food chain, reaching human beings or livestock leading to many diseases, seriously affecting human health, food safety and the development of livestock husbandry (Fu and Wang 2011). Soil heavy metal pollution has attracted increasing attention and microbial remediation of heavy metals has become a research hotspot in recent years. The mechanisms used for the microbial treatment of heavy metals can be divided into extracellular enrichment and precipitation, transformation through cell surface adsorption and precipitation, and transformation through intracellular adsorption and precipitation (Wang and Chen 2006). Therefore, heavy metal ions can be reduced or even lose their toxicity through cell metabolites or chemical groups on the cell surface or the cell itself, alone or through a combination of phenomena including adsorption, precipitation and valence transformation, as shown in Fig. 3.

In Table 1, we listed a number of relevant reports on the removal of heavy metals using microorganisms. Among them, *Deinococcus* has been studied for the remediation of heavy metal pollution because of its high resistance, tolerance to heavy metal ions and absorption and degradation ability. Chauhan et al. (2017) isolated the arsenic- and radiation-resistant *Deinococcus* DR1 from wetlands in northern India. After sequencing, the heavy metal resistance genes were identified by BLAST analysis. These included arsB, arsR and arsenate reductase genes, as well as outer membrane protein genes. In addition, there are heavy metal translocation P-type ATPase, heavy metal transport/detoxification proteins, heavy metal-related domain proteins and so on. This study has important practical value for bioremediation of heavy metal pollution. As can be seen in Table 1, *Deinococcus* has great potential and application value for dealing with pollution caused by heavy metal ions, but related technologies need to be further optimized and integrated.

**Table 1:** Treatment of heavy metal ions using various microorganisms

Microorganism	Heavy metal	pH	Temperature (°C)	Time (h)	Initial concentration	Results	Referencess
*Saccharomyces cerevisiae*	Pb(II)	5.0	25	2.0	50 mg/L	Monolayer adsorption capacity: 181.82 mg/g	Li et al. (2014)
*Saccharomyces cerevisiae*	Cu(II) Pb(II)	5.5 5.0	22 22	4.0 4.0	10–180 mg/L 10–180 mg/L	29.9 mg/g 72.5 mg/g	Amirnia et al. (2015)
*Amanita rubescens*	Pb(II) Cd(II)	5.0 5.0	20 20	0.5 0.5	10 mg/L 10 mg/L	38.4 mg/g 27.3 mg/g	Sari and Tuzen (2009)
*Micrococcus luteus*	Cd(II)	6.0	20	4.0	1000 μmol/L	444 ± 15 μmol/g	Machalová et al. (2015)
*Rhodopseudomonas palustris*	Cd(II)	6.0	20	4.0	1000 μmol/L	381 ± 1 μmol/g	Machalová et al. (2015)
*Pichia pastoris*	Zn(II)	5.0	28	48	0.5 mmol/L	Removal rate: 85%	Li et al. (2016)
*Trichoderma*	Cu(II)	5.0	30	2.0	100 mg/L	12.43 mg/g	Wei and Ting (2014)
*Microbacterium*	Cd(II)	7.0	N.A.	0.5	N.A.	15.6 mg/g	Park and Chon (2016)
*Arthrobacter* ps-5	Cu(II) Pb(II) Cr(VI)	5.0 5.5 6.0	N.A.	N.A.	10 mg/L	169.15 mg/g 216.09 mg/g 84.47 mg/g	Ye et al. (2014)
*Pseudomonas*	Hg(II)	N.A.	30	24	10 μM	Removal rate: 91%	Giovanella et al. (2017)
*D. radiodurans*	U(VI)	N.A.	30	504	235.5 μM	Removal rate: 89%	Fredrickson et al. (2000)
*Deinococcus Deino*-*phoN*-*yieF)*	U(VI)	5.0	30	13	1 mM	Removal rate: 77.6 ± 2.6%	Xu et al. (2018a, b, c)
*D. radiodurans*	U(VI)	5.0	Room temperature	3	1 mM	Removal rate: 90.0%	Misra et al. (2012)
*D. radiodurans*	U(VI)	5.0	25	6	0.8 mM	Removal rate: 90.0%	Appukuttan et al. (2006)
*D. radiodurans*	^60^Co	5.8	37	1.5	8.5 nM	Removal rate: > 60.0%	Gogada et al. (2015)
*D. radiodurans*	U(VI)	5.0	N.A.	6 408	1 mM 20 mM	260 mg/g 5.7 g/g	Appukuttan et al. (2011)

*N.A.* not available

#### Trehalose enhances stress resistance

The increase of trehalose content can improve the radiation tolerance of microbial cells (Nery et al. 2008). Our team found that the resistance of isolated yeast-like strains was related to their trehalose content (Liu et al. 2017). The researchers constructed engineering strains *APtps1*, *AP∆ath1* and *APT∆A* by over-expressing the trehalose-6-phosphate synthase gene *tps1* and knocking out the acid trehalase gene *ath1*. The results show that the *APT∆A* double mutant exhibit a survival rate of 1% under 20 kGy of gamma-radiation, 2% survival rate at a UV dosage of 250 J/m^2^, and tolerance up to 1500 mg/L Pb^2+^, which was in agreement with the high accumulation of intracellular trehalose compared to the wild-type strain (Liu et al. 2017). To improve the oxidative stress tolerance of *Clostridium tyrobutyricum* CCTCC W428, the gene *treS* (Jiang et al. 2013) was introduced into and expressed in *C. tyrobutyricum* (Wu et al. 2017). *Propionibacterium acidipropionici* accumulates high levels of trehalose during fermentation, especially under acidic stress. Through the genomic sequence analysis of *P. acidipropionici*, it was found that there are two putative trehalose synthesis pathways (OtsA-OtsB and TreY-TreZ), and an enhanced trehalose synthesis mutant was obtained by overexpression of the *otsA* gene encoding the OtsA-OtsB pathway enzyme. In this mutant, the fed-batch fermentation method has a maximum concentration of propionic acid reached 135 ± 6.5 g/L (Jiang et al. 2015).

The whole genome sequence of the strain R12 revealed that it contained two trehalose synthesis pathways and the trehalose synthase gene with a molecular weight of about 1700 bp was cloned and expressed, yielding a protein of about 66 kDa (Xu et al. 2013; Wang et al. 2009). One of the pathways is the trehalose synthase (TreS) pathway and the other is the maltose oligosaccharide synthase (TreY) pathway, which lays the foundation for subsequent experiments. A novel TreS gene identified from a metagenomic library (*Deinococcus sp.*) of saline-alkali soil are overexpressed in *E. coli* and purified, exhibiting its optimal activity condition at pH 9.0 and 45 °C, and tolerating most common metal ions (1 or 30 mM) except for Zn^2+^ and Hg^2+^ (Jiang et al. 2013). The catalytic efficiency (K_cat_/K_m_) of recombinant TreS to maltose was 4.1 times that of trehalose and at a relatively high maltose concentration (30%), the highest conversion rate of the conversion of maltose to trehalose by the TreS method is over 78% (Jiang et al. 2013). A TreS from *D. radiodurans* (DSMZ 20539) could maintain 56% of maximum activity after 8 h at 50° C and two recombinant trehalose synthases from *D. geothermalis* (DSMZ 11300) had a higher K_m_ value of 254 mM in comparison with the wild-type TreS, which indicated that TreS from *Deinococcus* has a similar features with TreS from others and may obtain further evidence for the production of TreS identified from *Deinococcus* (Filipkowski et al. 2012; Panek et al. 2013).

#### Other applications

The hydroxyl tetraterpenoid deoxyxanthine (DX) from *Deinococcus* can be used to synthesize functionalized gold nanoparticles (DX-AuNPs) through biotransformation, and this functionalized nanoparticles can induce the production of ROS in cancer cells by upregulating the expression of certain genes, thus leading to the apoptosis of cancer cells (Tian et al. 2018). IrrE is a specific protein that regulates the differential expression of genes that are closely related to biosynthesis, biofilm formation, transcriptional regulation and glucose metabolism. The expression of the *irrE* gene from *Deinococcus* in *Pseudomonas aeruginosa* that was seeded into microbial fuel cells (MFCs) significantly increased the substrate utilization, stress tolerance and bioelectricity generation capacity, and the cells achieved a power density that was 71% higher than the control value (Luo et al. 2018). *Deinococcus* has at least two surface proteins, Hpi and SlpA, whereby Hip is a highly efficient surface localization protein, and SlpA is associated with peptidoglycan. When a fusion protein composed of Hpi and endogenous PhoN was expressed in engineered *D. radiodurans* R1, it was localized in the membrane-bound fraction of the engineering bacteria and exhibited phosphatase activity in vivo and in vitro. The expression of synthetic phytochelatin (*EC*20) and cyanobacterial metallothionein (*smt*A) genes in *D. radiodurans* R1 was found to enhance its tolerance to and bioaccumulation of Cd^2+^. A DR1 strain carrying the former gene exhibited 2.5-fold higher tolerance to Cd^2+^ and 1.5-fold higher accumulation of Cd^2+^ than the control, while expressing the latter in DR1 led to a 2.5-fold increase of tolerance to Cd^2+^ compared to DR1 expressing *EC*20 (Chaturvedi and Archana 2014). Colored biofilms formed by *D. geothermalis* can potentially be used in paper mills because *D. geothermalis* has a strong resistance to ROS caused by IR, extreme pH, desiccation, solubilizing detergents and biocides and displays persistence against cleaning and chemical treatments (Peltola et al. 2008, 2011; Rasimus et al. 2011). It has a significant value for the development of surface display systems for the genus *Deinococcus* and their application in catalysis, environmental protection, biosensors, live vaccines, peptide libraries and other fields. *Deinococcus* contain many significant and even more still unknown genes waiting to be exploited.

## Conclusions and prospects

Extremophilic microorganisms can survive under harsh conditions such as cold, high temperatures, acid, alkali, drought and radiation. *Deinococcus radiodurans* is an extremely radiation-resistant microorganism that is present in various environments. It has significant resistance to desiccation, oxidative stress, ionizing radiation and ultraviolet radiation. The gene expression regulation mechanisms of *Deinococcus* under stress conditions, many of which are not yet clear, are being studied intensively. This bacterium is different from ordinary bacteria. Its high stress resistance makes it more widely applicable in various fields, such as dealing with soil and water polluted by radiation and heavy metals. Moreover, introducing its stress resistance genes into other microorganisms by genetic engineering can be used to improve their stress resistance, increase their scope of application and improve their production efficiency. The *pprI* gene has been introduced into *E. coli* and rapeseed, and it enhanced the resistance of these organisms to harsh environments such as high osmotic stress and drought. *Deinococcus* has great potential in saline-alkali land treatment, recovery and utilization of radioactive elements and precious metals, as well as the prevention and treatment of cancer caused by radiation. There are radiation hazards everywhere in modern life, such as electronic products and medical radiation treatment. Since *Deinococcus* is so strong, we can use it to develop novel radioprotectants or perhaps even to make skin care products against the damage caused by radiation or the oxygen radicals produced during normal aging. This is a certainly a development direction with great economic potential.

## Supplementary information

**Additional file 1: Table S1.** Strains of the genus *Deinococcus.*

## Data Availability

Not applicable.

## Abbreviations

*D. radiodurans*: *Deinococcus radiodurans*
DNA: deoxyribonucleic acid
ORFs: open reading frames
*D. wulumuqiensis*: *Deinococcus wulumuqiensis*
*E. coli*: *Escherichia coli*
bp: base pair
UV: ultraviolet ray
UVC: ultraviolet C
pH: potential of hydrogen
IR: ionizing radiation
ROS: reactive oxygen species
HR: homologous recombination
SSB: single-stranded DNA binding protein
ESDSA: Extended Synthesis-Dependent Strand Annealing
ssDNA: single-strand DNA
BER: base excision repair
NER: nucleotide excision repair
UVER: UV damage endonuclease (UvsE)-dependent excision repair
CPDs: cyclobutane pyrimidine dimers
MMR: DNA mismatch repair
UDGs: uracil-DNA glycosylases
RNA: ribonucleic acid
CDP: cytosine 5′-diphosphate
CTP: cytosine 5′-triphosphate
RNAP: RNA polymerase
CAT: catalases
SOD: superoxide dismutases
Dps: DNA protection during starvation
cAMP-CRP: cyclic adenosine monophosphate-cAMP receptor protein
TPS: trehalose-6-phosphate synthase
TPP: trehalose-6-phosphatase
MTHase: maltosyltrehalose hydrolase
MTSase: maltosyltrehalose synthase
DX: deoxyxanthine
MFCs: microbial fuel cells
*D. geothermalis*: *Deinococcus geothermalis*
