# Supplementary material for: The diversity and commonalities of the radiation-resistance mechanisms of Deinococcus and its up-to-date applications
Source: AMB Express. 2019 Sep 3;9:138. doi: 10.1186/s13568-019-0862-x (PMC6722170; doi:10.1186/s13568-019-0862-x)
Supplement: Supplementary file 1 — Additional file 1: Table S1. Strains of the genus Deinococcus. [file 13568_2019_862_MOESM1_ESM.doc]

**Table S1** Strains of the genus *Deinococcus*

| **Species** | **Strain** | **Sequence accession no. (16S rRNA )** | **Characteristics** | **Sources** | **References** |
| --- | --- | --- | --- | --- | --- |
| ***Deinococcus actinosclerus*** | BM2 = KEMB 5401-184 = JCM 30700 | KT448814 | Gram-positive, catalase and oxidase positive, strong resistance to gamma radiation, D10 value is 9 kGy, but UVC resistance is weak. | Rocky hillside soil | Joo et al. (2016) |
| ***Deinococcus aerius*** | TR0125 = DSM 21212 = JCM 11750 | AB087288 | Orange colonies, non-motile, high GC content, strongly resistant to desiccation, UV and γ-radiation. | Upper atmosphere dust | Yang et al. (2009) |
| ***Deinococcus aerolatus*** | 5516T-9 = JCM 15442 = KACC 12745 | EU622978 | Pink colonies, Gram-positive, aerobic, G+C content is 61.0%. | Air | Yoo et al. (2010) |
| ***Deinococcus aerophilus*** | 5516T-11 = JCM 15443 = KACC 12746 | EU622979 | Pink colonies, Gram-positive, aerobic, G+C content is 59.3%. | Air | Yoo et al. (2010) |
| ***Deinococcus aetherius*** | ST0316 = DSM 21230 = JCM 11751 | AB087287 | Pink colonies, non-motile, aerobic, G+C content is 69.8l%, resistant to desiccation, UV and γ-radiation. | Stratospheric dust | Yang et al. (2010) |
| ***Deinococcus alpinitundrae*** | ME-04-04-52 = LMG 24283 = NRRL B-41948 | EF635408 | Psychrophilic, aerobic, optimum temperature is 10°C, pH is 6-9, G+C content is 62.6%, can withstand 4 kGy of γ-radiation | Soil | Callegan et al. (2008) |
| ***Deinococcus altitudinis*** | ME-04-01-32 = LMG 24022 = NRRL B-41947 | EF635407 | Psychrophilic, aerobic, optimum temperature is 10°C, pH is 6-9，G+C content is 65.9%, can withstand 4 kGy of γ-radiation. | Soil | Callegan et al. (2008) |
| ***Deinococcus claudionis*** | PO-04-19-125 = LMG 24282 = NRRL B-41949 | EF635406 | Psychrophilic, aerobic, optimum temperature is 10°C, pH is 6-9, G+C content is 63.1%, can withstand 4 kGy of γ-radiation. | Soil | Callegan et al. (2008) |
| ***Deinococcus radiomollis*** | PO-04-20-132 = LMG 24019 = NRRL B-41950 | EF635404 | Psychrophilic, aerobic, optimum temperature is 10°C, pH is 6-9, G+C content is 63.2%, can withstand 4 kGy of γ-radiation. | Soil | Callegan et al. (2008) |
| ***Deinococcus antarcticus*** | G3-6-20 = DSM 27864 = CCTCC AB 2013263 | KC494323 | Pink colonies, non-motile, aerobic, G+C content is 63.l%, resistant to desiccation and UV. | Soil | Dong et al. (2015) |
| ***Deinococcus apachensis*** | KR-36 = LMG 22135 = NRRL B-23948 | AY743264 | Pink colonies, non-motile, aerobic, G+C content is 68.5%, Gram-positive, optimum temperature is 30°C, oxidase and catalase positive. | Soil | Rainey et al. (2005) |
| ***Deinococcus hohokamensis*** | KR-40 = LMG 22129 = NRRL B-23949 | AY743256 | Pink colonies, non-motile, aerobic, G+C content is 67.9%, Gram-positive, optimum temperature is 30°C, oxidase and catalase positive. | Soil | Rainey et al. (2005) |
| ***Deinococcus hopiensis*** | KR-140 = LMG 22133 = NRRL B-23943 | AY743262 | Pink colonies, non-motile, aerobic, G+C content is 66.2%, Gram-positive, optimum temperature is 30°C, oxidase and catalase positive. | Soil | Rainey et al. (2005) |
| ***Deinococcus maricopensis*** | LB-34 = DSM 21211 = LMG 22137 = NRRL B-23946 | CP002454 | Pink colonies, non-motile, aerobic, G+C content is 71.1%, Gram-positive, optimum temperature is 40°C, oxidase and catalase positive. | Soil | Rainey et al. (2005) |
| ***Deinococcus navajonensis*** | KR-114 = LMG 22131 = NRRL B-23951 | AY743259 | Pink colonies, non-motile, aerobic, G+C content is 66.4%, Gram-positive, optimum temperature is 30°C, oxidase positive and catalase negative. | Soil | Rainey et al. (2005) |
| ***Deinococcus papagonensis*** | KR-241 = LMG 22139 = NRRL B-23961 | AY743280 | Pink colonies, non-motile, aerobic, G+C content is 69.0%, Gram-positive, optimum temperature is 30°C, oxidase and catalase positive. | Soil | Rainey et al. (2005) |
| ***Deinococcus pimensis*** | KR-235 = LMG 22244 = NRRL B-23994 | AY743277 | Pink colonies, non-motile, aerobic, G+C content is 71.5%, Gram-positive, optimum temperature is 30°C, catalase positive and cytochrome oxidase negative. | Soil | Rainey et al. (2005) |
| ***Deinococcus sonorensis*** | KR-87 = LMG 22172 = NRRL B-23941 | AY743283 | Pink colonies, non-motile, aerobic, G+C content is 70.3%, Gram-positive, optimum temperature is 30°C, oxidase and catalase positive. | Soil | Rainey et al. (2005) |
| ***Deinococcus yavapaiensis*** | KR-236 = LMG 22171 = NRRL B-23960 | AY743279 | Deep pink and red colonies, non-motile, aerobic, G+C content is 66.1%, Gram-positive, optimum temperature is 30°C, oxidase and catalase positive. | Soil | Rainey et al. (2005) |
| ***Deinococcus aquaticus*** | PB314 = KCTC 12552 = NBRC 101311 | DQ017708 | Gram-negative, non-spore forming, galactosidase and catalase positive, hydrolysis of starch was positive, tolerates 3% w/v) NaCl, G+C content is 68.3%. | Fresh water | Im et al. (2008) |
| ***Deinococcus caeni*** | Ho-08 = KCTC 12553 = NBRC 101312 | DQ017709 | Gram-negative, non-spore forming, galactosidase and catalase positive, hydrolysis of starch was positive, tolerate 3% w/v) NaCl, G+C content is 68.4%. | Activated sludge from wastewater treatment | Im et al. (2008) |
| ***Deinococcus aquatilis*** | CCM 7524 = CCUG 53370 | AM940971 | Light pink colonies, non-spore-forming, optimum temperature is 30°C, good alkali resistance, pH value up to 11. | Water | Kämpfer et al. (2008) |
| ***Deinococcus aquiradiocola*** | TDMA-uv53 = CCUG 53612 = JCM 14371 = NBRC 102118 | AB265180 | Light pink colonies, resistant to desiccation, UV. Gram-positive, non-spore-forming, G+C content is 69.1%. | Radiation-contaminated environment | Asker et al. (2009) |
| ***Deinococcus arenae*** | SA1 = JCM 31047 = KCTC 33741 | KF790633 | Gram-negative, red colonies, aerobic, optimum temperature is 30°C, pH 7.0, catalase positive, oxidase negative, G+C content is 69.5%. | Beach sand | Lee et al. (2016) |
| ***Deinococcus soli*** | N5 = JCM 19176 = KCTC 33153 | KC771032 | Gram-positive, resistant to over 4 kGy of γ-radiation, G+C content is 71.4 %. | Paddy soil | Cha et al. (2014) |
| ***Deinococcus budaensis*** | FeSTC15-38 = NCAIM B.02630 = DSM 101791 | LN864925 | Gram-negative, optimum pH is 7.0 and the optimum temperature is 28°C, G+C content is 68.2 %. | Hot spring | Makk et al. (2016) |
| ***Deinococcus carri*** | MA1003 = KACC 17965 = NBRC 110142 | JX089327 | Gram-positive, non-spore forming, non-motile, pH 6.5, optimum temperature is 28°C, catalase and oxidase positive, resistant to UV, G+C content is 76.0 %. | The air conditioning system of a car | Kim et al. (2015) |
| ***Deinococcus metallilatus*** | MA1002 = KACC 17964 = NBRC 110141 | JX089329 | Gram-positive, non-spore forming, non-motile, pH 6.5, optimum temperature is 40°C, catalase and oxidase positive, resistant to UV, G+C content is 70.5%. | The air conditioning system of a car | Kim et al. (2015) |
| ***Deinococcus cellulosilyticus*** | 5516J-15 = DSM 18568 = KACC 11606 | DQ883809 | Pink colonies, resistant to UV, catalase and oxidase positive, G+C content is 61.3%. | Air | Weon et al. (2007) |
| ***Deinococcus citri*** | NCCP-154 = JCM 19024 = DSM 24791 = KCTC 13793 | AB558498 | Gram-positive, aerobic, optimum temperature is 30°C, pH 7.0, G+C content is 70.0 %. | Citrus leaf ulcer lesion | Ahmed et al. (2014) |
| ***Deinococcus daejeonensis*** | MJ27 = JCM 16918 = KCTC 13751 | JF806527 | Gram-positive, aerobic, red colonies, G+C content is 67.6 %, tolerates 0–4% w/v) NaCl, D_10_ value of resistance to γ-radiation is over 9 kGy. | Sludge of wastewater treatment | Srinivasan et al. (2012) |
| ***Deinococcus depolymerans*** | TDMA-24 = CCUG 53609 = JCM 14368 = NBRC 102115 | AB264134 | Gram-positive, red colonies, non-motile, aerobic, catalase, oxidase and β-galactosidase positive, G+C content is 70.5-70.6%. | Fresh water | Asker et al. (2011) |
| ***Deinococcus deserti*** | VCD115 = DSM 17065 = LMG 22923 | CP001114 | Gram-negative, resistant to UV and γ-radiation, white colonies, aerobic, protease and catalase positive, G+C content is 59.8%. | Desert | De et al. (2005) |
| ***Deinococcus erythromyxa*** | UWO 1045 = ATCC 187 = DSM 11630 = IFO now NBRC) 15344 = JCM 12416 | Y11330 | Red colonies, contains many carotenoids, aerobic, tolerates 5% w/v) NaCl, catalase positive, G+C content is 71.0%. | Air | Brooks et al. (1980) |
| ***Deinococcus proteolyticus*** | Kobatake strain MRP = ATCC 35074 = CCM 2703 = DSM 20540 = HAMBI 2066 = IFO now NBRC) 15345 = JCM 6276 = NBRC 101906 = UWO 1056 = VKM Ac-1939 | CP002536 | Orange-red smooth colonies, the cell wall has at least three layers, has multiple carotenoids, strictly aerobic, tolerates 1% w/v) NaCl, catalase positive, the G+C content is 65.0 %. | Feces | Brooks et al. (1980) |
| ***Deinococcus radiodurans*** | ATCC 13939= CCUG 27074 = CIP 104750 = DSM 20539 = HAMBI 1924 = IFO now NBRC) 15346 = JCM 16871 = LMG 4051 = UWO 288 = VKM B-1422 | Y11332 | Red smooth colonies, contains multiple carotenoids, strictly aerobic, tolerates γ-radiation of 1.5 Mrad and UV radiation of 1800 ergs·cm^-2^ per second for 10 minutes, tolerates 1% w/v) NaCl and can grow in the presence of 5% w/v) NaCl, G+C content is 70.0%. | Corrupt canned meat | Brooks et al. (1980) |
| ***Deinococcus radiophilus*** | ATCC 27603= DSM 20551 = IFO now NBRC) 15347 = JCM 21311 = NCTC 10785 = UWO 1055 = VKM B-1468 | Y11333 | Orange-red smooth colonies, the cell wall has at least three layers, has multiple carotenoids, strictly aerobic, tolerates 5% w/v) NaCl, catalase positive, the G+C content is 62.0 %, tolerate 1.5 Mrad of γ-radiation. | Irradiated fish | Brooks et al. (1980) |
| ***Deinococcus radiopugnans*** | ATCC 19172 = DSM 12027 = IFO now NBRC) 15348 = JCM 21312 = JCM 16872 = UWO 293 | Y11334 | Orange-red smooth colonies, the cell wall has at least three layers, has multiple carotenoids, strictly aerobic, tolerates 1% w/v) NaCl, catalase positive, tolerates 1.5 Mrad of γ-radiation. | Haddock | Brooks et al. (1980) |
| ***Deinococcus ficus*** | CC-FR2-10 = CCUG 51391 = CIP 108832 | AY941086 | Lightly colored, smooth colonies, Gram-positive, aerobic, oxidase positive, resistant to UV. | Rhizosphere of Fig Tree | Lai et al. (2006) |
| ***Deinococcus frigens*** | AA-692 = DSM 12807 = NRRL B-41046 | AJ585981 | Orange or pink, smooth colonies, psychrotrophic, aerobic or facultatively anaerobic, growth temperature is 9-18°C, pH 3.8-9.0, tolerates 0-10% w/v) NaCl, the G+C content is 61.8-65.5%. | Antarctic soil and rock | Hirsch et al. (2004) |
| ***Deinococcus marmoris*** | AA-63 = DSM 12784 = NRRL B-41042 | AJ585986 | Orange or pink colonies, psychrotrophic, aerobic, growth temperature is 9-16°C, lipase and amylase negative, G+C content is 61.3-62.8%. | Antarctic soil and rock | Hirsch et al. (2004) |
| ***Deinococcus saxicola*** | AA-1444 = DSM 15974 = NRRL B-41043 | AJ585984 | Orange or pink colonies, psychrotrophic, aerobic, optimum temperature is 9.0°C, pH 4-9, negative for nitrate reduction and β-galactosidase, lipase and amylase positive, G+C content is 59.5%. | Antarctic soil and rock | Hirsch et al. (2004) |
| ***Deinococcus mumbaiensis*** | CON-1 = DSM 17424 = MTCC 7297 | DQ003135 | Gram-negative, light pink colonies, aerobic, G+C content is 70.0%. | Contaminated tryptone glucose yeast agar medium | Shashidhar and Bandekar (2006) |
| ***Deinococcus geothermalis*** | AG-3a = CIP 105573 = DSM 11300 | CP000359 | Gram-positive, orange colonies, optimum temperature is 47°C, pH 6.5, oxidase and catalase positive, α-galactosidase negative and β-galactosidase positive, G+C content is 65.9%. | Hot spring | Ferreira et al. (1997) |
| ***Deinococcus murrayi*** | ALT-1b = CIP 105574 = DSM 11303 | Y13041 | Gram-positive, orange colonies, optimum temperature is 47°C, pH 8.0, oxidase and catalase positive, α-galactosidase and β-galactosidase positive, G+C content is 69.9%. | Hot spring | Ferreira et al. (1997) |
| ***Deinococcus gobiensis*** | I-0 = CGMCC 1.7299 = DSM 21396 = JCM 16679 | EU427464 | Red colonies, facultatively anaerobic, catalase and oxidase positive, urease positive, G+C content is 65.4%, tolerates 15 kGy of γ-radiation and 600 J·m^-2^ of UV. | Desert | Yuan et al. (2009) |
| ***Deinococcus grandis*** | KS 0485 = ATCC 43672= DSM 3963 = HAMBI 2161 = IAM 13005 = JCM 6269 | Y11329 | Gram-negative, red or pink colonies, catalase and DNAse positive, urease and oxidase negative, G+C content is 69.0%, resistant to 1.0 Mrad of γ-radiation, growth temperature is 37-42°C. | Animal droppings and freshwater fish | Rainey et al. (1997) |
| ***Deinococcus humi*** | MK03 = JCM 17915 = KCTC 13619 | GQ339889 | Gram-positive, red colonies, strictly aerobic, G+C content is 64.5%, resistant to 9 kGy of γ-radiation, tolerates 0-3% w/v) NaCl. | soil | Srinivasan et al. (2012) |
| ***Deinococcus indicus*** | Wt/1a = DSM 15307 = MTCC 4913 | AJ549111 | Gram-negative, has the ability to resist arsenic and radiation, red colonies, non-motile, G+C content is 65.8%, resistant to 5.87 J·cm^-2^ of UV. | Arsenic contaminated water | Suresh et al. (2004) |
| ***Deinococcus knuensis*** | 16F3H = KCTC 33794 = JCM 31406 | LC148307 | Gram-negative, non-motile, aerobic, tolerates 4% w/v) NaCl, resistant to UV, G+C content is 65.7%. | River | Lee et al. (2017) |
| ***Deinococcus puniceus*** | DY1 = JCM 18576 = KCTC 33027 | JQ991920 | Gram-positive, red colonies, resistant to γ-radiation, oxidase negative and catalase positive, G+C content is 58.7%. | Radiated soil | Lee et al. (2015) |
| ***Deinococcus sedimenti*** | 16F1L= KCTC 33796 = JCM 31405 | KU758885 | Gram-positive, non-motile, pink colonies, optimum temperature is 25°C, resistant to over 2 kGy of γ-radiation, G+C content is 67.2%. | Han River | Lee et al. (2016) |
| ***Deinococcus metalli*** | 1PNM-19 = GIMCC 1.654 = CCTCC AB 2014198 = DSM 27521T | JQ608330 | Gram-positive, aerobic, orange colonies, resistant to UV, G+C content is 71.7±0.1%. | Lead-zinc ore | Feng et al. (2015) |
| ***Deinococcus misasensis*** | TDMA-25 = CCUG 53610 = JCM 14369 = NBRC 102116 | AB264135 | Gram-positive, red or pink colonies, strictly aerobic, resistant to γ-radiation and UV, oxidase and catalase positive, optimum temperature is 40-45°C, pH 7.0-8.0, G+C content is 53.5%. | Fresh water | Asker et al. (2008) |
| ***Deinococcus roseus*** | TDMA-uv51 = CCUG 53611 = JCM 14370 = NBRC 102117 | AB264136 | Gram-positive, red or pink colonies, strictly aerobic, resistant to γ-radiation and UV, oxidase and catalase positive, optimum temperature is 30-35°C, pH 7.0-8.0, G+C content is 57.4%. | Fresh water | Asker et al. (2008) |
| ***Deinococcus peraridilitoris*** | KR-200 = CIP 109416 = LMG 22246 | EF141348 | Aerobic, resistant to 10 kGy of γ-radiation, G+C content is 63.9 mol%. | Coastal desert dry soil | Rainey et al. (2007) |
| ***Deinococcus persicinus*** | KSY3-6 = KCTC 33787 = JCM 31313 | KU865686 | Gram-negative, aerobic, optimum temperature is 25°C, catalase positive and oxidase negative, resistant to γ-radiation and UV, G+C content is 62.0%. | Soil | Jeon et al. (2016) |
| ***Deinococcus phoenicis*** | 1P10ME = NRRL B-59546 = DSM 27173 | EU977832 | Gram-positive, light pink colonies, aerobic, resistant to 8 kGy of γ-radiation. | Cleanroom at Kennedy Space Center | Vaishampayan et al. (2014) |
| ***Deinococcus piscis*** | 3ax = DSM 19767 = MTCC9123 | DQ683348 | Gram-positive, aerobic, optimum temperature is 35°C, pH 7.0, catalase and oxidase positive, amylase, urease, tryptophan deaminase and β-galactosidase negative, resistant to 7.4 kGy of γ-radiation. | Marine fish | Shashidhar and Bandekar (2009) |
| ***Deinococcus radiotolerans*** | C1 = KCTC 33150 = JCM 19173 | KC771028 | Gram-negative, pink colonies, growth temperature is15–42°C, pH 6–11, tolerates 0–2% w/v) NaCl, G+C content is 68.6%, resistant to 8 kGy of γ-radiation. | Soil | Cha et al. (2014) |
| ***Deinococcus swuensis*** | DY59 = KCTC 33033 = JCM 18581 | JQ991923 | Gram-positive, optimum temperature is 30°C, tolerates 3% w/v) NaCl, resistant to 5 kGy of γ-radiation, G+C content is 66.5%. | Soil | Lee et al. (2013) |
| ***Deinococcus reticulitermitis*** | TM-1 = CGMCC 1.10218 = NBRC 106334 | HM214546 | Gram-negative, red colonies, resistant to γ-radiation and UV, β-galactosidase positive, optimum temperature is 20-40°C, pH 6.0-10.0, G+C content is 65.6%. | Intestinal tract of wood termites | Chen et al. (2011) |
| ***Deinococcus ruber*** | JSH3-1 = KCTC 33790 = JCM 31311 | KU865686 | Gram-positive, aerobic, resistant to γ-radiation and UV, growth temperature is 4-37°C, pH 7.0, G+C content is 62.0%, oxidase and catalase positive. | Garden soil | Kim et al. (2017) |
| ***Deinococcus saudiensis*** | YIM F302 = CGMCC 1.15089 = DSM 29933 | KT278764 | Gram-negative, pink colonies, aerobic, resistant to γ-radiation and UV, growth temperature is 15-45°C, pH 6.0-8.0, G+C content is 69.3%. | Desert | Hussain et al. (2016) |
| ***Deinococcus seoulensis*** | 16F1E = JCM 31404 = KCTC 33793 | LC148306 | Gram-positive, red colony, G+C content is 66.9%, growth temperature is 10-37°C, pH 4-10, tolerate 5% w/v) NaCl, resistant to γ-radiation. | Han River | Lee et al. (2016) |
| ***Deinococcus wulumuqiensis*** | R12 = CGMCC 1.8884 = NBRC 105665 | EU025028 | Gram-positive, orange colonies aerobic, optimum temperature is 37°C, pH 7.0-8.0, resistant to 10 kGy of γ-radiation and 700 J·m^-2^ of UV, G+C content is 66.7%. | Soil contaminated by radiation in Northwestern China | Wang et al. (2010) |
| ***Deinococcus xibeiensis*** | R13 = CGMCC 1.8885 = NRBC 105666 | FJ439568 | Gram-positive, pink colonies, aerobic, optimum temperature is 30°C, pH 7.0, resistant to 10 kGy of γ-radiation and 700 J·m^-2^ of UV, G+C content is 63.8%. | Soil contaminated by radiation in Northwest China | Wang et al. (2010) |
| ***Deinococcus xinjiangensis*** | X-82 = CCTCC AB 207226 = NRRL B-51287 | EU626561 | Gram-positive, light yellow colonies, resistant to UV, optimum temperature is 30°C, pH 7.0, G+C content is 60.0%. | Soil | Peng et al. (2009) |
| ***Deinococcus yunweiensis*** | YIM 007 = DSM 17005 = KCTC 3962 | DQ344634 | Gram-negative, red colonies, optimum temperature is 30°C, pH 7.0-7.5, G+C content is 64.1%, resistant to 16 kGy of γ-radiation and 624 J·m^-2^ of UV. | An agar plate from Yunnan Institute of Microbiology | Zhang et al. (2007) |

**References**

Ahmed I, Abbas S, Kudo T, Iqbal M, Fujiwara T, Ohkuma M (2014) *Deinococcus citri* sp. nov. isolated from citrus leaf canker lesions. Int J Syst Evol Micr 64:4134-4140.

Asker D, Awad TS, Beppu T, Ueda K (2008) *Deinococcus misasensis* and *Deinococcus roseus*, novel members of the genus *Deinococcus*, isolated from a radioactive site in Japan. Syst Appl Microbiol 31:43-49.

Asker D, Awad TS, Beppu T, Ueda K (2009) *Deinococcus aquiradiocola* sp. nov. isolated from a radioactive site in Japan. Int J Syst Evol Micr 59:144-149.

Asker D, Awad TS, Mclandsborough L, Beppu T, Ueda K (2011) *Deinococcus depolymerans* sp. nov. a gamma- and UV-radiation-resistant bacterium, isolated from a naturally radioactive site. Int J Syst Evol Micr 61:1448-1453.

Brooks BW, Murray RGE, Johnson JL, Stackebrandt E, Woese CR, Fox GE (1980) Red-pigmented micrococci: a basis for taxonomy. Int J Syst Evol Micr 30:627-646.

Callegan RP, Nobre MF, McTernan PM, Battista JR, Navarro-Gonzalez R, Mckay CP, da Costa MS, Rainey FA (2008) Description of four novel psychrophilic, ionizing radiation-sensitive *Deinococcus* species from alpine environments. Int J Syst Evol Micr 58:1252-1258.

Cha S, Srinivasan S, Seo T, Kim MH (2014) *Deinococcus radiotolerans* sp. nov. a gamma-radiation-resistant bacterium isolated from gamma ray-irradiated soil. Antonie Van Leeuwenhoek 105:229-235.

Cha S, Srinivasan S, Seo T, Kim MK (2014) *Deinococcus soli* sp. nov. a gamma-radiation-resistant bacterium isolated from rice field soil. Curr Microbiol 68:777-783.

Chen W, Wang B, Hong H, Yang H, Liu SJ (2011) *Deinococcus reticulitermitis* sp. nov. isolated from a termite gut. Int J Syst Evol Micr 62:78-83.

De GA, Chapon V, Servant P, Christen R, Fischer-Le Saux M, Sommer S, Heulin T (2005) *Deinococcus deserti* sp. nov. a gamma-radiation-tolerant bacterium isolated from the Sahara Desert. Int J Syst Evol Micr 55:2441-2446.

Dong N, Li HR, Yuan M, Zhang XH, Yu Y (2015) *Deinococcus antarcticus* sp. nov. isolated from soil. Int J Syst Evol Micr 65:331-335.

Feng GD, Yang SZ, Xiong X, Li HP, Zhu HH (2015) *Deinococcus metalli* sp. nov. isolated from an abandoned lead-zinc mine. Int J Syst Evol Microbiol 65:3457-3461.

Ferreira AC, Nobre MF, Rainey FA, Silva MT, Wait R, Burghardt J, Chung AP, daCosta MS (1997) *Deinococcus geothermalis* sp. nov. and *Deinococcus murrayi* sp. nov. two extremely radiation-resistant and slightly thermophilic species from hot springs. Int J Syst Bacteriol 47:939-947.

Hirsch P, Gallikowski CA, Siebert J, Peissl K, Kroppenstedt R, Schumann P, Stackebrandt E, Anderson R (2004) *Deinococcus frigens* sp. nov. *Deinococcus saxicola* sp. nov. and *Deinococcus marmoris* sp. nov. low temperature and draught-tolerating, UV-resistant bacteria from continental Antarctica. Syst Appl Microbiol 27:636-645.

Hussain F, Khan IU, Habib N, Xian WD, Hozzein WN, Zhang ZD, Zhi XY, Li WJ (2016) *Deinococcus saudiensis* sp. nov. isolated from desert. Int J Syst Evol Microbiol 66:5106-5111.

Im WT, Jung HM, Ten LN, Kim MK, Bora N, Goodfellow M, Lim S, Jung J, Lee ST (2008) *Deinococcus aquaticus* sp. nov. isolated from fresh water, and *Deinococcus caeni* sp. nov. isolated from activated sludge. Int J Syst Evol Micr 58:2348-2353.

Jeon SH, Kang MS, Joo FS, Kim FB, Lim S, Jeong SW, Jung HY, Srinivasan S, Kim MK (2016) *Deinococcus persicinus* sp. nov. a radiation-resistant bacterium from soil. Int J Syst Evol Micr 66:5077-5082.

Joo ES, Lee JJ, Kang MS, Lim S, Jeong SW, Kim EB, Jeon SH, Srinivasan S, Kim MK (2016) *Deinococcus actinosclerus* sp. nov., a novel bacterium isolated from soil of a rocky hillside. Int J Syst Evol Micr 66:1003-1008.

Kämpfer P, Lodders N, Huber B, Falsen E, Busse HJ (2008) *Deinococcus aquatilis* sp. nov. isolated from water. Int J Syst Evol Micr 58:2803-2806.

Kim DU, Lee H, Lee JH, Ahn JH, Lim S, Jeong S, Park SY, Seong CN, Ka JO (2015) *Deinococcus metallilatus* sp. nov. and *Deinococcus carri* sp. nov. isolated from a car air-conditioning system. Int J Syst Evol Micr 65:3175-3182.

Kim EB, Kang MS, Joo ES, Jeon SH, Jeong SW, Lim SY, Jung HY, Srinivasan S, Kim MK (2017) *Deinococcus ruber* sp. nov. a radiation-resistant bacteria isolated from a soil. Int J Syst Evol Microbiol 67:72-76.

Lai WA, Kampfer P, Arun AB, Shen FT, Huber B, Rekha PD, Young CC (2006) *Deinococcus ficus* sp. nov. isolated from the rhizosphere of Ficus religiosa L. Int J Syst Evol Micr 56:787-791.

Lee D, Cha S, Jang JH, Seo T (2016) *Deinococcus arenae* sp. nov. a novel species isolated from sand in South Korea. Antonie Van Leeuwenhoek 109:1055-1062.

Lee JJ, Lee HJ, Jang GS, Yu JM, Kim MK (2013) *Deinococcus swuensis*, sp. nov. a gamma-radiation-resistant bacterium isolated from soil. J Microbiol 51:305-311.

Lee JJ, Lee YH, Park SJ, Lee SY, Park S, Lee DS, Kang IK, Ten LN, Jung HY (2017) *Deinococcus knuensis*, sp. nov. a bacterium isolated from river water. Antonie Van Leeuwenhoek 110:407-414.

Lee JJ, Lee YH, Park SJ, Lim S, Jeong SW, Lee SY, Park S, Choi HW, Kim MK, Jung HY (2016) *Deinococcus sedimenti,* sp. nov. isolated from river sediment. J Microbiol 54:802-808.

Lee JJ, Lee YH, Park SJ, Lim S, Jeong SW, Lee SY, Cho YJ, Kim MK, Jung HY (2016) *Deinococcus seoulensis* sp. nov. a bacterium isolated from sediment at Han River in Seoul, Republic of Korea. J Microbiol 54:537-542.

Lee JJ, Srinivasan S, Lim S, Joe M, Im S, Kim MK (2015) *Deinococcus puniceus* sp. nov. a Bacterium Isolated from Soil-Irradiated Gamma Radiation. Curr Microbiol 70:464-469.

Makk J, Tóth EM, Anda D, Pál S, Borsodi AK (2016) *Deinococcus budaensis* sp. nov. a mesophilic species isolated from biofilm sample of a hydrothermal spring cave. Int J Syst Evol Micr 66:5345-5351.

Peng F, Zhang L, Luo X, Dai J, Fang C (2009) *Deinococcus xinjiangensis* sp. nov. isolated from desert soil. Int J Syst Evol Micr 59:709-713.

Rainey FA, Ferreira M, Nobre MF, Ray K, Bagaley D, Earl AM, Battista JR, Gomez-Silva B, McKay CP, da Costa MS (2007) *Deinococcus peraridilitoris* sp. nov. isolated from a coastal desert. Int J Syst Evol Micr 57:1408-1412.

Rainey FA, Nobre MF, Schumann P, Stackebrandt E, da Costa MS (1997) Phylogenetic diversity of the *Deinococci* as determined by 16S ribosomal DNA sequence comparison. Int J Syst Bacteriol 47:510-514.

Rainey FA, Ray K, Ferreira M, Gatz BZ, Nobre MF, Bagaley D, Rash BA, Park MJ, Earl AM, Shank NC (2005) Extensive diversity of ionizing-radiation-resistant bacteria recovered from Sonoran Desert soil and description of nine new species of the genus *Deinococcus* obtained from a single soil sample. Appl Environ Microb 71:5225-5235.

Shashidhar R, Bandekar JR (2006) *Deinococcus mumbaiensis* sp. nov. a radiation-resistant pleomorphic bacterium isolated from Mumbai, India. FEMS Microbiol Lett 254:275-280.

Shashidhar R, Bandekar JR (2009) *Deinococcus piscis* sp. nov. a radiation-resistant bacterium isolated from a marine fish. Int J Syst Evol Micr 59:2714-2717.

Srinivasan S, Kim MK, Lim S, Joe M, Lee M (2012) *Deinococcus daejeonensis* sp. nov., isolated from sludge in a sewage disposal plant. Int J Syst Evol Micr 62:1265-1270.

Srinivasan S, Lee JJ, Lim S, Joe M, Kim MK (2012) *Deinococcus humi* sp. nov., isolated from soil. Int J Syst Evol Micr 62:2844-2850.

Suresh K, Prabagaran SR., Sengupta S, Shivaji S (2004) *Deinococcus indicus* sp. nov. an arsenic-resistant bacterium isolated from an aquifer in West Bengal, India. Int J Syst Evol Micr 54:457-461.

Vaishampayan P, Roberts AH, Augustus A, Pukall R, Schumann P, Schwendner P, Mayilraj S, Salmassi T, Venkateswaran K (2014) *Deinococcus phoenicis* sp. nov. an extreme ionizing-radiation-resistant bacterium isolated from the Phoenix Lander assembly facility. Int J Syst Evol Micr 64:3441-3446.

Wang W, Mao J, Zhang Z, Tang Q, Goodfellow M (2010) *Deinococcus wulumuqiensis* sp. nov. and *Deinococcus xibeiensis* sp. nov. isolated from radiation-polluted soil. Int J Syst Evol Micr 60:2006-2010.

Weon HY, Kim BY, Schumann P, Son JA, Jang J, Go SJ, Kwon SW (2007) *Deinococcus cellulosilyticus* sp. nov. isolated from air. Int J Syst Evol Micr 57:1685-1688.

Yang Y, Itoh T, Yokobori S, Itahashi S, Shimada H, Satoh K, Ohba H, Narumi I, Yamagishi A *(*2009) *Deinococcus aerius* sp. nov. isolated from the high atmosphere. Int J Syst Evol Micr 59:1862-1866.

Yang Y, Itoh T, Yokobori S, Shimada H, Itahashi S, Satoh K, Ohba H, Narumi I, Yamagishi A (2010) *Deinococcus aetherius* sp. nov. isolated from the stratosphere. Int J Syst Evol Micr 60:776-779.

Yoo SH, Weon HY, Kim SJ, Kim YS, Kim BY, Kwon SW (2010) *Deinococcus aerolatus* sp. nov. and *Deinococcus aerophilus* sp. nov. isolated from air samples. Int J Syst Evol Micr 60:1191-1195.

Yuan M, Zhang W, Dai S, Wu J, Lin M (2009) *Deinococcus gobiensis* sp. nov. an extremely radiation-resistant bacterium. Int J Syst Evol Micr 59:1513-1517.

Zhang YQ, Sun CH, Li WJ, Yu LY, Zhou JQ, Zhang YQ, Xu LH, Jiang CL (2007) *Deinococcus yunweiensis* sp. nov. a gamma- and UV-radiation-resistant bacterium from China. Int J Syst Evol Micr 57:370-375.
